# Analytical methods and experimental approaches for electrophysiological studies of brain oscillations

**DOI:** 10.1016/j.jneumeth.2014.03.007

**Published:** 2014-05-15

**Authors:** Joachim Gross

**Affiliations:** Institute for Neuroscience and Psychology, University of Glasgow, Glasgow, UK

**Keywords:** Oscillations, MEG, EEG, Spectral analysis, Single-trial, Information theory, Classification

## Abstract

Brain oscillations are increasingly the subject of electrophysiological studies probing their role in the functioning and dysfunction of the human brain. In recent years this research area has seen rapid and significant changes in the experimental approaches and analysis methods. This article reviews these developments and provides a structured overview of experimental approaches, spectral analysis techniques and methods to establish relationships between brain oscillations and behaviour.

## Highlights

• Review of methods for studying brain oscillations with MEG/EEG.
• Covers experimental approaches and analytical methods.
• Focus on novel experimental approaches such as entrainment.
• Describes methods for identifying relation between brain oscillations and behaviour.

## 1 Introduction

In recent years the investigation of brain oscillations has received increasing interest. Many studies have demonstrated that specific cognitive processes are reflected in brain oscillations with characteristic temporal, spatial and spectral signatures (Siegel et al., 2012; Schnitzler and Gross, 2005; Wang, 2010; Thut et al., 2012). These studies support the view that brain oscillations are directly related to computational processes implementing fundamental information processing tasks in the human brain. Several recent reviews provide a very good overview of this aspect of the field (Siegel et al., 2012; Schnitzler and Gross, 2005; Wang, 2010; Giraud and Poeppel, 2012; Arnal and Giraud, 2012; Singer, 2013; Thut et al., 2012; Lopes da Silva, 2013; Jerbi et al., 2009).

Instead, this review presents and discusses the available analytical methods to study brain oscillations. It aims to give an overview of the relevant approaches and methods for studying brain oscillations as well as providing information for making informed decisions about the suitable methods for a given data analysis. As such this review is hopefully of interest for novices as well as more experienced researchers in the field of brain oscillations.

The interested reader is referred to previous methodological reviews of this and related fields. For example, Greenblatt and co-workers have recently reviewed connectivity measures (Greenblatt et al., 2012). These measures are often applied in the context of studies investigating brain oscillations. Other reviews have focused on a comparison of methods for spectral analysis (Muthuswamy and Thakor, 1998; Bruns, 2004; Wacker and Witte, 2013; Roach and Mathalon, 2008; Worrell et al., 2012). The current review complements the existing literature by reviewing recent developments in experimental approaches for studying brain oscillations, by discussing the different stages of spectral analysis for electrophysiological data and by providing an overview of analytical techniques for the identification of relationships between brain oscillations and behaviour.

## 2 Identification of brain oscillation

**Fig. 1:**
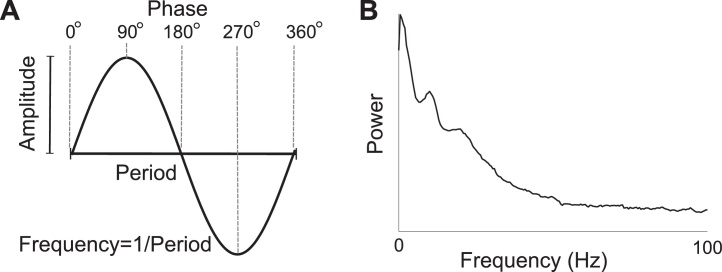
(A) An oscillation is characterised by its amplitude and frequency. A specific point within an oscillatory cycle is unambiguously identified by the phase irrespective of the frequency of the oscillation. (B) Power spectrum of a 5-min MEG signal recorded from an occipital sensor of a participant in the absence of any instructed task. The spectrum shows the typical 1/*f* pattern with a peak at around 10 Hz corresponding to ongoing alpha oscillations.

Physicists have studied oscillations for a long time. They emerge in many dynamical systems. A well-known example is the pendulum. Its dynamics can be approximated by a simple differential equation and its deflection from rest position over time is described by a sine function. The simple harmonic oscillator is therefore unambiguously specified by the amplitude, frequency and phase (Fig. 1A). However, identification and characterisation of oscillations in electrophysiological data is considerably more complicated.

Fig. 1B shows a power spectrum from 5 min resting data for an MEG channel over occipital areas. Typical alpha oscillations appear as a peak in the power spectrum at a frequency of about 10 Hz. Still, the spectrum is dominated by low frequency components and shows the typical 1/*f* characteristic. Closer examination reveals that these neural oscillations are non-stationary and show fluctuations in amplitude and frequency. In addition, rhythmic components can exist in the signal that are not purely oscillatory in nature. Examples include the rolandic mu-rhythm with a comb-like signal shape (Gastaut, 1952) and steady-state signals induced by rhythmic trains of stimuli (Picton et al., 2003). However, the most commonly used spectral analysis techniques do not account for these non-stationarities. All this makes identification of ongoing oscillations challenging and even more so for oscillations that are less prominent than the relatively strong alpha oscillations.

In a typical experiment researchers aim to identify stimulus-related or task-related modulations of brain oscillations by examining amplitude changes of oscillations over time and frequency. But under what conditions are amplitude modulations in the time-frequency domain considered sufficient evidence for the modulation of an intrinsic brain oscillation? Some commonly observed effects in a time-frequency spectral analysis are transient and others cover a large frequency band. For example, a typical evoked response will be represented as a short phase-locked power increase at low frequencies (≤10 Hz) and cannot be considered an oscillation per se. Other effects can be observed in frequency bands where the respective brain area might not show ongoing oscillations at rest. Unfortunately, no established criteria exist for the identification of brain oscillations or for classifying spectral perturbations as caused by intrinsic brain oscillations. But some of the criteria recently suggested for the identification of entrainment effects can also be useful to help address this question (Thut et al., 2011a, Box 2).

## 3 Experimental approaches to studying brain oscillations

Brain oscillations can be studied using a variety of experimental approaches some of which have received increasing interest over the last few years. Here, I will make an attempt to provide a structured overview of these approaches, describe their merit and highlight some successful applications. I distinguish event-related and continuous approaches that are described in separate sections.

### 3.1 Event-related paradigms

Event-related experiments typically study the modulation of brain oscillations time-locked to an event. In the simplest case the event will be the presentation of a stimulus of a short duration or a short movement such as a button press. The aim of studies using this approach is typically the identification of significant modulations in the amplitude of brain oscillations (e.g. Siegel et al., 2012; Capilla et al., 2014; Gross et al., 2007; Cheyne, 2013; Neuper et al., 2006). The approach can be extended by introducing several conditions. Still, the underlying assumption in these studies is that brain oscillations return to a constant baseline level before the start of the next event. Researchers try to assure that this is the case by using temporal delays between stimuli in the order of about 2–10 s. However, it is known that brain oscillations are modulated by the general behavioural state of participants (Steriade, 2000), attention (Klimesch, 2012; Thut et al., 2012), expectation (Rohenkohl and Nobre, 2011; Capilla et al., 2014; Thut et al., 2006) and prediction (Arnal and Giraud, 2012). Therefore, ‘baseline activity’ especially in blocked experimental designs will possibly be biased by experimentally induced changes in arousal, attention or expectancy.

Although this experimental approach has been typically used to study the modulation of the amplitude of oscillations it can as well be applied to study effects of phase. For example, Schyns and colleagues instructed participants to perform categorisation of emotional faces and used Information Theory to establish phase modulations that code specific features of the presented stimuli (Schyns et al., 2011).

Another type of experiments uses this experimental approach to study the effect of spontaneous variations in ongoing brain oscillations on stimulus processing. A typical example is the repeated presentation of a near-threshold stimulus. During analysis data epochs are sorted according to behavioural outcome (e.g. perceived or not perceived) to identify significant differences in neural activity before presentation of the stimulus. This approach has been used by van Dijk et al. to demonstrate that variation in the amplitude of parieto-occipital alpha oscillations at the time of stimulus presentation predict performance in a visual discrimination task (van Dijk et al., 2008). Similarly, Thut et al. reported a correlation between prestimulus occipital alpha power and reaction time in a covert visuo-spatial attention task (Thut et al., 2006). Extending the approach to the study of oscillatory phase effects is straightforward (Vanrullen and Dubois, 2011; Busch et al., 2009; Mathewson et al., 2009). Interestingly, recent evidence indicates that perception is not only modulated by local prestimulus power and phase but also by the prestimulus network state characterised by connectivity measures (Weisz et al., 2014; Hanslmayr et al., 2007).

This event-related approach lends itself to pharmacological intervention to test the effect of neuromodulators on cognitive processing (Bauer et al., 2012). To further examine the causality of effects of variations in brain oscillations on behavioural performance real-time analysis of MEG/EEG activity can be used to trigger stimulus presentation at times of pre-defined brain states (e.g. high or low alpha power) (Jensen et al., 2011).

Interestingly, this approach can be generalised beyond sensory pathways by replacing sensory stimuli with TMS stimuli while simultaneously measuring EEG signals. TMS-evoked EEG signals can be examined for oscillatory components that can reflect resonance frequencies of the stimulated thalamo-cortical system (Rosanova et al., 2009; Garcia et al., 2011).

### 3.2 Continuous paradigms

In contrast to event-related paradigms continuous paradigms do not rely primarily on temporally defined events (although these can be later defined during analysis, see e.g. Gross et al., 2013b). Analysis of data recorded with continuous paradigms often discards temporal information and focuses on spectral information and their experimentally induced changes.

Different classes of continuous paradigms can be distinguished. First, neural activity can be studied in the absence of any task and any controlled stimulation. These ‘resting-state’ studies often aim at identifying functional or effective connectivity pattern (Brookes et al., 2011; Hipp et al., 2012; de Pasquale et al., 2010, 2012). They have been successful in demonstrating the existence of consistent long-range amplitude correlations particularly at frequencies below 30 Hz despite the fact that the alleged ‘resting state’ is ill defined and difficult to control. Electrophysiological resting-state data has recently been used for clinical studies (Vecchio et al., 2013; Uhlhaas and Singer, 2012; Stam, 2010).

A second class of continuous paradigms uses one or several continuous tasks that can last several minutes. For example continuous isometric contraction or simple motor tasks have been used in healthy participants (Jerbi et al., 2007; Gross et al., 2002) and in patients (Timmermann et al., 2003; Schnitzler and Gross, 2005; Pollok et al., 2009). Typically, these studies aim at the identification of spectral signal components that are significantly different between conditions or correlate with behavioural performance. Other studies have used continuous stimulation with naturalistic stimuli to demonstrate that brain oscillations support sensory coding and task-dependent communication (Gross et al., 2013b; Honey et al., 2012; Morillon et al., 2010; Betti et al., 2013; Ng et al., 2013).

Entrainment paradigms can be considered as a third class of continuous paradigms. They are defined by rhythmic features in the stimulus. These could be trains of short stimuli with a constant SOA (Cravo et al., 2013; Capilla et al., 2011; Lakatos et al., 2008; Herrmann, 2001) or continuous rhythmic modulations of features of the stimulus (such as amplitude or frequency (Henry and Obleser, 2012; Picton et al., 2003)). Some naturalistic stimuli (such as continuous speech) contain quasi-rhythmic components that entrain brain oscillations (Gross et al., 2013b; Zion Golumbic et al., 2013; Luo et al., 2010). Investigating the mechanisms and dynamics of this entrainment improves our understanding of the role that brain oscillations play in segmenting and coding continuous sensory stimuli (Giraud and Poeppel, 2012).

The entrainment approach has been extended to neurostimulation techniques such as TMS or TACS. Recent studies suggest that this approach allows a controlled modulation of brain oscillations independent of sensory stimulation (Thut et al., 2011a,b; Helfrich et al., 2014; Chanes et al., 2013).

Entrainment studies play an increasingly important role for establishing a causal role of brain oscillations for cognitive processes. Traditionally, the oscillation–behaviour relationship has been established by means of correlation or by contrasting oscillations between experimental conditions showing different behavioural performance. In contrast, entrainment studies attempt to modulate brain oscillations so that consequences for behavioural performance can be studied in a controlled manner.

## 4 Spectral analysis

Spectral analysis provides the neuroscientists with a set of tools to study oscillatory components in neural time series.

The main motivation for this analysis is typically one of the following

• identifying oscillatory components in neural time series;
• identifying dependencies between oscillations in different brain areas (connectivity);
• identifying dependencies between oscillations (their frequency, amplitude or phase) and cognitive processes or behaviour.

Spectral analysis typically relies on the transformation of time series into the frequency domain. In the frequency-domain signals are represented as a linear combination of oscillatory basis functions. Because of the use of oscillatory basis functions the frequency-domain representation is optimal (and sparse) for signals with oscillatory components and facilitates the identification of these components and their modulations over time. However, as briefly discussed in the introduction, the interpretation of spectral analysis results of electrophysiological data is far from trivial since the analysis has to account for the time-varying and sometimes transient nature of physiological oscillations. In addition, MEG/EEG data is often contaminated by (rhythmic) artefacts that will negatively affect spectral representations (such as power spectra or time-frequency representations). In this section we, first, review analytical techniques that prepare the data for spectral analysis. Then, we highlight different techniques that perform the required transformation of time-series data into the frequency domain. For a more formal discussion of spectral analysis we recommend the use of more specialised literature (Stoica and Moses, 1997; Flandrin, 1999; Brillinger, 2001; Percival and Walden, 1993).

### 4.1 Data preprocessing

This short section mainly covers aspects related to the preprocessing of data that are specifically important for the analysis of brain oscillations. In general, standard procedures should be used for removing artefacts from the data (Gross et al., 2013a; Picton et al., 2000; Keil et al., 2014). For example, data needs to be inspected for discontinuities (e.g. Squid jumps in MEG data). Filtering these artefacts may lead to filter ringing and can generate oscillatory components in the filtered data. However, for the purpose of studying brain oscillations particular consideration should be given to rhythmic artefacts. We can distinguish between physiological and non-physiological artefacts. An example for a non-physiological artefact is magnetic/electric noise generated by air-conditioning equipment. These artefacts can have rhythmic components in the range of physiological brain activity (10–40 Hz). These can be detected by spectral analysis of empty-room recordings. One prominent source of rhythmic physiological activity is the heart. Especially MEG magnetometer devices are sensitive to these cardiac artefacts.

Several analytical tools exist that allow for the correction of these artefacts. These artefact correction methods mostly make use of linear transformations or regression techniques applied to the sensor data (Wallstrom et al., 2004; Parra et al., 2005; Ille et al., 2002; Schlögl et al., 2007). Linear transformations can be obtained from principal component analysis (PCA), independent component analysis (ICA) (Parra et al., 2005; Ille et al., 2002; Onton and Makeig, 2006; Rong and Contreras-Vidal, 2006; Barbati et al., 2004; Jung et al., 2000), or through signal space projection (SSP) (Uusitalo and Ilmoniemi, 1997) or signal space separation (SSS) (Taulu et al., 2004; Taulu and Simola, 2006). PCA, ICA and regression techniques rely on the assumption that the data space can be divided into a signal space and an artefact/noise space and that this separation is valid for all time points in the data. PCA-based techniques require an artefact template to estimate the artefact/noise space whereas ICA provides an automatic separation of the data into statistically independent components. The topography, time course and spectral characteristics of these components are used for manual or (semi-) automatic assignment of components to the signal or artefact space. SSS performs the separation of the signal and noise subspaces based on spherical harmonics expansion (Nolte, 2003) of the data making use of the fact that the physiological activity is generated within the spatial compartment enclosed by the MEG sensors.

### 4.2 Transformation into frequency domain

This section provides a short overview of different analytical techniques for the transformation of data into the frequency domain. In principle, the researcher has to decide between parametric and non-parametric methods. Examples of non-parametric methods are the Fourier transform, wavelet transform or the Hilbert transform. The most commonly used parametric spectral estimation technique is based on autoregressive (AR) modelling where a time series is expressed as a linear combination of past values and a noise term. Although spectra computed from AR-models can potentially result in higher time and/or frequency resolution (Nalatore and Rangarajan, 2009) they are sensitive to parameters such as the model order. Spectral analysis using AR-models is extensively covered elsewhere (Astolfi et al., 2008; Schloegl et al., 2006; Seth et al., 2011).

Controlling the time-frequency resolution is probably the most critical factor for time-frequency analysis. There is a fundamental trade-off between time and frequency resolution that is formalised in the Heisenberg uncertainty principle. The upper bound of the joint time-frequency resolution is achieved by Morlet (or Gabor) Wavelets. Interestingly, this type of wavelets can be used in the context of Fourier analysis, wavelet analysis or the Hilbert transform.

In the following we cover the most commonly used analytical techniques for spectral analysis. Other tutorials and reviews are available that cover more practical aspects of this analysis (Herrmann et al., 2013; Gross et al., 2013b; Roach and Mathalon, 2008).

#### 4.2.1 Fourier analysis

Here and in the following sections we consider the case of a time-frequency analysis of MEG/EEG data over multiple trials (computation of a power spectrum can be seen as a special case of this). A Fourier-based time-frequency analysis typically uses the short-time Fourier method. Here, a short segment of data is selected from the first trial. Data points in this segment are weighted by a tapering function and subjected to the Fourier transform (often using the computationally efficient Fast Fourier Transform (FFT)). Tapering (e.g. using a Hanning window) is recommended because it reduces spectral leakage. The result is a complex spectrum in the frequency domain. Then, the next segment of data is selected (often with a large overlap with the previous window) and the computation is repeated. In this way, the data is transformed into the frequency domain in short segments for all the trials. Finally, the absolute value (or square of the absolute value) is averaged across trials for each time window separately leading to a time-frequency spectrum. It should be noted that the length *L* of the data segment determines the spectral frequency resolution (1/*L*). To some extent the frequency resolution can be increased by zero-padding (adding zeros to the selected data segment increases *L* and thereby the frequency resolution). For recommended settings we refer the interested reader to existing tutorials and guidelines (Herrmann et al., 2013; Gross et al., 2013b; Roach and Mathalon, 2008; Keil et al., 2014).

Instead of a single taper (such as Hanning window) multiple orthogonal tapers can be used to reduce the variance of the spectral estimate and to achieve a controlled smoothing in the frequency domain that is independent of the spectral resolution (Percival and Walden, 1993; Mitra and Pesaran, 1999). This technique, termed multi-tapering, is particularly suited for the analysis of high frequency oscillations that typically show a large frequency jitter because it effectively integrates spectral power over a range of frequencies.

#### 4.2.2 Wavelet analysis

Wavelet analysis is based on a set of oscillatory basis functions that are constructed from a prototypical ‘mother wavelet’. One of the most commonly used wavelets is the Morlet wavelet (or Gabor wavelet) that has an optimal trade-off between time and frequency resolution. It is constructed from a complex-valued wave weighted with a Gaussian function.

Changing the length of the wavelet can control the trade-off between time and frequency resolution. A longer wavelet will improve the frequency resolution but reduce the temporal resolution. All wavelets are derived from the mother wavelet by compression in time. To compute a time-frequency spectrum the time series are convolved with the wavelets leading to complex-valued spectral estimates.

Whereas the length of the data segment is typically constant in Fourier-based spectral analysis the length of the wavelet decreases for higher frequencies. The changing wavelet length leads to a frequency-adaptive temporal-spectral resolution of the wavelet spectral estimates. High frequencies have a better temporal resolution compared to low frequencies.

#### 4.2.3 Hilbert–Huang transform

The Hilbert–Huang transform has been recently proposed for the computation of time-frequency spectra from nonlinear and non-stationary processes (Huang et al., 1998). It is based on a combination of Empirical Mode Decomposition (EMD) and the Hilbert transform.

In contrast to wavelet and Fourier analysis it uses adaptive basis functions (called intrinsic mode functions, IMF) that are derived from the data. An IMF can change amplitude and frequency over time. The subsequently applied Hilbert transform leads to an estimate of instantaneous frequency and amplitude for each IMF. These estimates are then combined across IMFs into the Hilbert–Huang spectrum. EMD alone or as part of the Hilbert–Huang transform has been used to study for example the generation of event-related or TMS-induced EEG changes (Burgess, 2012; Pigorini et al., 2011; Liang et al., 2005).

#### 4.2.4 Hilbert transform

The Hilbert transform on its own is a useful tool for spectral analysis (Freeman, 2007). It transforms a time-series into the analytic signal that allows computation of instantaneous phase and amplitude. However, phase and amplitude estimates are only interpretable for narrow-band signals. Since brain signals can consist of multiple independent oscillatory components at different frequencies band-pass filtering is required before applying the Hilbert transform (Pikovsky et al., 2003; Wacker and Witte, 2013). If the phase is used for further analysis it is important to use filters that do not lead to phase distortion. One suitable choice is the forward and reverse filtering with an FIR filter (see also Widmann and Schröger, 2012). In principle a whole time-frequency representation can be constructed based on the Hilbert transform by using a bank of bandpass-filters and hilbert-transforming the filtered signal. Although the extracted amplitude or phase signal has the sampling frequency of the original data it should be noted that the time-frequency resolution of the estimated amplitude signal is determined by the properties of the bandpass filter. In general, a wider passband will allow for more rapid amplitude fluctuations in the Hilbert-transformed signal.

#### 4.2.5 Matching pursuit analysis

Matching pursuit (MP) is an algorithm that decomposes a time series into a sum of ‘atoms’ from a pre-defined dictionary (Mallat and Zhang, 1993). This dictionary can consist of Morlet wavelets to optimise the time-frequency trade-off (Wacker and Witte, 2011) but can also include additional functions that can capture non-oscillatory components in the data. Interestingly, the dictionary can contain wavelets of different length even for the same frequency and the MP algorithm selects the wavelet that best matches the signal. In practice, the inner product between the respective signal segment and all atoms in the dictionary is computed and the projection of the signal on the atom with the largest inner product is subtracted. In the next iteration the process is repeated with the residual signal. A time-frequency representation can be computed by linear superposition of the time-frequency representation of the individual atoms.

The use of this over-complete dictionary has interesting and relevant applications as it allows to differentiate between transient and oscillatory components (Ray and Maunsell, 2011). A transient (non-oscillatory) signal component will have the largest inner product with a non-oscillatory atom whereas an oscillatory component extending over several cycles will be best represented with an oscillatory atom of matching frequency and duration. In comparison, it is very difficult to differentiate between transient and oscillatory signal components in time-frequency representations based on Fourier or wavelet analysis. Non-oscillatory signal components will be represented by a linear combination of oscillatory basis functions. A short, transient signal component will be represented in wavelet or Fourier analysis by a power increase over a broad frequency band and could be interpreted as an ‘oscillatory’ phenomenon. Moreover, the phase and amplitude of this representation will be correlated over time and can lead to spurious results in cross-frequency analysis (Kramer et al., 2008).

Due to its data-adaptive nature the MP algorithm leads to time-frequency spectra with high time and frequency resolution. A possible drawback lies in the fact that MP algorithms consist of a finite set of atoms and therefore introduce an estimation bias towards these atoms (although this can be addressed with stochastic dictionaries (Durka et al., 2001) or dictionary learning (Barthélemy et al., 2013)).

#### 4.2.6 Comparison of time-frequency methods

It has been shown that the STFT, Wavelet-, and Hilbert approach are essentially equivalent and can be seen as a convolution of the original time series with a complex kernel (Bruns, 2004; Le Van Quyen et al., 2001; Kiebel et al., 2005). Identical or at least very similar results can be obtained with all three methods by an appropriate selection of parameters. An important consideration is the time-frequency resolution across different frequency bands. In a typical Fourier analysis the time-frequency resolution is kept constant whereas it is frequency-dependent in a typical wavelet analysis. Some degree of frequency-dependence of the time-frequency resolution is desirable in most cases because standard definitions of functional frequency bands of brain oscillations (based on clinical observations and task-related modulations in electrophysiological studies) show a logarithmic organisation (Buzsaki and Draguhn, 2004) highlighting the importance of a higher frequency resolution for low frequencies and a lower frequency resolution for higher frequencies (Gross et al., 2013b).

Ray et al. provide an interesting comparison of STFT and MP methods (Ray et al., 2008, Suppl. Discussion 3). They demonstrate that non-stationarity can lead to signal cancellations in Fourier analysis but not in MP analysis. This effect is most pronounced for high frequencies.

An elegant and rather comprehensive overview and comparison of time-frequency methods can be found in Wacker and Witte (2013). Wacker and colleagues point out that the optimality of time-frequency results depends on the matching between the signal components and the basis functions of the respective time-frequency method. Unfortunately, the time-frequency characteristics of components of the MEG/EEG signal are unknown and the a priori choice of time-frequency resolution (by selecting a window length for Fourier analysis or the wavelet length for wavelet analysis) will likely lead to suboptimal results. It is therefore recommended to perform time-frequency analysis with different time-frequency resolutions. Another promising approach is the use of matching-pursuit (MP) techniques where the appropriate time-frequency resolution is adaptively determined by the algorithm (Wacker and Witte, 2013).

## 5 Post-processing of spectral data

Time-frequency analysis with any of the techniques described in the previous section is typically performed on single trials and leads to a complex-valued representation of each trial in the time-frequency plane. Various measures can be derived from this representation (Roach and Mathalon, 2008). The two fundamental measures are amplitude and phase. Amplitude is computed as the absolute value (or magnitude) of the complex numbers. Sometimes power is used instead by computing the square of the amplitude. Phase is computed as the argument of the complex number (by computing the arctangent). Single-trial power and/or phase time-frequency data is then subjected to further analysis to identify significant relationships between these measures and behaviour.

### 5.1 Baseline correction

Whereas phase is a bounded measure (between 0 and 2 pi) amplitude typically requires normalisation to account for inter-individual differences and changes of ongoing oscillations over time. Often the modulation of ongoing oscillations time-locked to an event is of interest motivating a baseline correction. In addition, time-frequency power results are dominated by low frequencies because of the typical 1/*f* characteristic of power in MEG/EEG recordings. Appropriate baseline normalisation (e.g. when post stimulus power is expressed as relative change to baseline) can improve identification and visualisation of stimulus-induced effects in high frequencies.

However, there is little consensus on when and how to perform baseline correction. In fact, it is known that the amplitude of oscillations in the baseline window affects behavioural performance in a subsequent task (van Dijk et al., 2008; Hanslmayr et al., 2007; Romei et al., 2008) and oscillations in the baseline may be the very subject of a study. In principle, baseline correction can be performed in a number of ways. The most commonly used measures express post-stimulus power at each individual frequency as a difference, ratio, percent increase or in units of decibel or standard deviation (*z*-score) of the baseline power at that frequency.

Traditionally, this baseline correction is done after averaging the single trial power time-frequency results. However, a recent study (Grandchamp and Delorme, 2011) convincingly demonstrates the sensitivity of this classical baseline correction to noisy trials leading to an overestimation of post-stimulus power. Based on simulations and analysis of real data they conclude that single-trial normalisation using the full trial (instead of only the pre-stimulus baseline) outperforms both the classical baseline correction and also the single-trial normalisation based on the baseline window. Results were similar for different normalisations (relative change, decibel or in units of baseline standard deviation) with a slight advantage for the normalisation that uses the baseline standard deviation. Similarly, Hu et al. report an over-estimation of power when relative change normalisation is employed for baseline correction for individual subjects and especially if it is employed for single trials (Hu et al., 2013). This bias is absent for baseline normalisation based on subtracting mean baseline power per frequency. However, performance of different normalisation schemes will likely depend on the experimental paradigm and the time window selected for the single trials. It could be desirable to test various normalisation strategies for a given study.

### 5.2 Identifying significant differences between conditions

Time-frequency analysis is often motivated by the aim to identify significant differences of the amplitude of brain oscillations between two experimental conditions (or between post-stimulus time-windows and baseline). Both, parametric and non-parametric methods can be used to address this problem. Parametric methods make assumptions about the distribution of the tested data, which is not the case for non-parametric methods. However, if the assumptions are met parametric methods will always be as or more sensitive than non-parametric methods (Kiebel et al., 2005). And they allow interesting applications such as the deconvolution of time-frequency data into different components (Litvak et al., 2013) or multivariate analysis (Soto et al., 2009).

Over recent years non-parametric statistics has become more and more popular for the analysis of time-frequency data (Maris and Oostenveld, 2007; Pantazis et al., 2005). It can be flexibly used in a number of ways for different test statistics and on different types of data (e.g. on single trials for individual analysis or on single subjects for group analysis). In principle, a given test statistic (e.g. *t*-statistic between two conditions) is compared to a null-distribution. In the single subject case this null-distribution can be computed from multiple *t*-tests after a random assignment of trials to the two conditions. The multiple comparison problem (MCP) in the context of non-parametric statistics can be addressed in various ways. *P*-values can be corrected by the false discovery rate (FDR) method (Benjamini and Hochberg, 1995), by using cluster-level statistics (Maris and Oostenveld, 2007) or maximum statistics (Pantazis et al., 2005). Again, the clustering can be performed flexibly along different dimensions of the data space (e.g. across time-frequency for a single sensor, or across time-sensor for a given frequency, etc.).

An obvious (but relevant) variation of this condition-contrast approach is based on a sorting of trials according to behavioural outcome. A typical example is the presentation of a near-threshold target where analysis is based on the statistical contrast of detected versus undetected target (or fast versus slow reaction times, see e.g. van Dijk et al., 2008; Gross et al., 2007; Hanslmayr et al., 2007; Vanrullen et al., 2011; Mathewson et al., 2009; Reinhart et al., 2011).

Interestingly, this approach can also be applied for the analysis of oscillatory phase. It has been used to demonstrate that prestimulus oscillatory phase affects detection performance (Vanrullen et al., 2011; Mathewson et al., 2009). In this case trials are split into two conditions according to behavioural performance and the single-trial distributions of phase (computed from the complex values in the time-frequency plane) are compared between the two conditions for each time-frequency point. Established methods from circular statistics can be used to reveal significant differences between conditions (Fisher, 1995; Berens, 2009).

### 5.3 Identifying brain–behaviour correlations from time-frequency data

More sophisticated methods exist that go beyond a simple comparison of two conditions. Most of these methods make use of the single-trial time-frequency information and often relate this to single-trial behaviour (such as reaction time, subjective stimulus intensity, detection accuracy etc.). This section reviews analytical methods that can be used to relate single-trial time-frequency data to behaviour.

A straightforward extension of the two-condition approach is the computation of correlations between single-trial time-frequency data and behaviour (e.g. (Zhang et al., 2008; Tan et al., 2013; Reinhart et al., 2011) but see Rousselet and Pernet, 2012). For each time-frequency point the respective measure (power or phase) can be correlated across trials with behavioural performance (note that circular correlation has to be used for phase) leading to a time-frequency map of correlation.

The correlation approach can be easily extended to more complex models. General linear models (GLM) are often used to establish linear dependencies between brain activity and behaviour and have been applied to time-frequency data as well (Wyart et al., 2012; Luckhoo et al., 2012; Kiebel et al., 2005; Litvak et al., 2011; Pernet et al., 2011; Debener et al., 2005; Cohen and Cavanagh, 2011; Gould et al., 2012; Huang et al., 2013). For example, power at each time frequency point can be regressed on one or multiple variables (such as real or subjective stimulus intensity or reaction time) including their interactions (Cohen and Cavanagh, 2011). In their study Cohen and Cavanagh used robust regression because it is less sensitive to outliers (2011). Another study compared several regression models to investigate which oscillatory components predict the subjective experience of pain intensity (Schulz et al., 2011).

Given the high dimensionality of the spectral data (time, frequency, space) it can be advantageous to perform multivariate analysis to identify significant dependencies between brain activity and behaviour. Multivariate tests can be used to identify significant components (modes) in high-dimensional space (Carbonell et al., 2004; Friston et al., 1996; McIntosh and Lobaugh, 2004) and are in general more sensitive compared to univariate tests. However, univariate tests are required to localise significant effects within that space.

Partial least squares (PLS) is one of these multivariate tools that has been used in Neuroimaging (McIntosh and Lobaugh, 2004; Hu et al., 2013; Düzel et al., 2003). PLS can be used to decompose time-frequency data into components (or latent variables) that show correlations with behaviour or differences between experimental conditions. PLS analysis if often combined with randomisation statistics to identify significant correlations between latent variables and behaviour.

However, these GLM-based methods rely on the assumption of Gaussianity of the underlying data distribution and typically probe for linear relationships between time-frequency power and behaviour (although non-linear regressors can be used). Indeed, non-linear (e.g. parabolic) dependencies between oscillatory power and behavioural performance have been identified using a simple binning strategy (Linkenkaer-Hansen et al., 2004). Single-trials are sorted according to their power in a specific time-frequency window into a number of bins (e.g. 10 percentile bins). The corresponding behavioural measure is then averaged across trials for each bin. This analysis is performed on each individual subject and thereby accounts for inter-individual variation in oscillatory power. Finally, statistical analysis can be performed across subjects on the variation of the behavioural measure over bins (Capilla et al., 2014; Linkenkaer-Hansen et al., 2004).

An interesting method to detect and quantify non-linear dependencies between time-frequency data and behaviour that does not depend on the assumption of Gaussianity comes from Information Theory (Cover and Thomas, 2006). Mutual information quantifies the amount of information (in bits) that one random variable (e.g. oscillatory power) contains about another (e.g. behaviour) (Cover and Thomas, 2006; Quian Quiroga and Panzeri, 2009; Magri et al., 2009). It can be used as a versatile tool to study functional connectivity (Liu and Ioannides, 2006; Maruyama and Ioannides, 2010) or relate oscillatory measures to stimulus or behaviour (Schyns et al., 2011). Interestingly, phase and amplitude (and also their interactions) can be studied and compared within the same computational framework (see e.g. Schyns et al., 2011; Gross et al., 2013b).

Another multivariate analysis approach for time-frequency data is based on classification (Besserve et al., 2007; Pfurtscheller and Neuper, 2006) and often makes use of support vector machines (SVM) (Schulz et al., 2012), neural networks (Fuentemilla et al., 2010), regularised least squares classifier (Tsuchiya et al., 2008) or linear discriminant analysis (Kauppi et al., 2013; Lou et al., 2013). For example, Schulz and colleagues used SVM to predict pain perception in individual subjects from single-trial time-frequency data separately for each electrode (Schulz et al., 2012). Significance of classification was tested with permutation statistics. Tsuchiya et al. (2008) identified diagnostic information for distinguishing faces from geometric pattern from intracranial recordings across time, frequency and space. In addition to application of a regularised least squares classifier in the time-frequency domain they mapped the spatial distribution of informative time-frequency power by performing a searchlight decoding analysis. Fuentemilla and co-workers performed multivariate pattern classification on time-frequency data from MEG sensors to distinguish trials where either indoor or outdoor scenes had been visually presented to participants (Fuentemilla et al., 2010; Jafarpour et al., 2013). Application of the classifier during the maintenance period revealed memory replay that was coordinated by the phase of theta oscillations.

Similarly, representational similarity analysis (Kriegeskorte et al., 2008) can identify relations between behavioural data and time-frequency representations of electrophysiological data. Leske and co-workers combined this analysis with a searchlight approach to demonstrate that the strength of an auditory illusory percept correlates with the amplitude of alpha and beta oscillations predominantly in auditory areas (Leske et al., 2013).

## 6 Conclusion

Supported by developments over the last years the study of brain oscillations has changed into a relatively mature field of science with respect to the experimental approaches and analytical methods. Validated techniques exist for spectral analysis of MEG/EEG data. Most commonly, spectral analysis is performed by using Fourier-, Wavelet- or Hilbert transform. Relatively novel methods such as matching pursuit techniques appear to be promising and may soon find their way into the mainstream analysis pipelines.

Considerable progress has been made regarding the statistical analysis of the high-dimensional data that appropriately correct for multiple statistical comparisons. Amongst these methods, high-dimensional cluster analysis and FDR correction are routinely used. There is still considerable heterogeneity in the way brain–behaviour relations are established from spectral data. Beyond simple cases such as statistical contrast between conditions that are associated with different behaviour (or behavioural performance) there is little consensus on analytical methods or approaches. But a promising trend seems to be the move towards single-trial based analysis as researchers increasingly recognise that trial-by-trial variability in behaviour and neural signals is not only caused by noise but rather represents meaningful variations in brain state. Hopefully this trend will soon be complemented by taking into consideration inter-subject variability (Renvall et al., 2012; Schulz et al., 2011).

Furthermore, the recent developments reviewed here have led to a significantly improved understanding of brain oscillations. Specifically, we have learned about the importance of phase for neural communication and stimulus coding and processing, have developed protocols to modulate brain oscillations through pharmacological intervention, brain-state triggered stimulation, neurostimulation or sensory entrainment and have a variety of methods available to study the effect of changing brain states on stimulus processing.

These and ongoing developments will undoubtedly lead to more exciting discoveries about the intricate relationship between brain oscillations and human behaviour.
